# 20-Years of Population-Based Cancer Registration in Hepatitis B and Liver Cancer Prevention in The Gambia, West Africa

**DOI:** 10.1371/journal.pone.0075775

**Published:** 2013-09-30

**Authors:** Ebrima Bah, Maria Patrizia Carrieri, Pierre Hainaut, Yusupha Bah, Ousman Nyan, Makie Taal

**Affiliations:** 1 Ministry of Health and Social Welfare, Banjul, The Gambia; 2 Institut National de la Sante et de la recherche Medicale (INSERM) U379, Marseille, France; 3 International Prevention Research Institute (iPRI), Lyon, France; 4 National Cancer Registry, Fajara, The Gambia; 5 University of The Gambia, Brikama, The Gambia; 6 University of Tampere, Tampere, Finland; 7 The Gambia Hepatitis Intervention Study (GHIS) project, Fajara, The Gambia; University of Modena & Reggio Emilia, Italy

## Abstract

**Background:**

The Gambia Hepatitis Intervention Study (GHIS) was designed as a randomised control trial of infant hepatitis B vaccination applied to public health policy, with the main goal of preventing primary liver cancer later in adult life in The Gambia. To that effect, the National Cancer Registry of The Gambia (NCR), a population-based cancer registry (PBCR), was established in 1986 to actively collect data on all cancer diagnosis nation-wide. We extracted 20-years (1990-2009) of data to assess for the first time, the evolution of the most common cancers, also describe and demonstrate the role of the PBCR in a hepatitis B and liver cancer prevention programme in this population.

**Methods and Findings:**

We estimated Age-Standardised Incidence Rates (ASR (W)) of the most common cancers registered during the period by gender. The registration period was divided into four 5-year intervals and incidence rates were estimated for each interval. The most common cancers in males were liver, prostate, lung plus bronchus, non-Hodgkin lymphoma (NHL) and stomach, accounting for 60%, 5%, 4%, 5% and 3%, respectively. Similarly, cancers of the cervix uteri, liver, breast and NHL, were the most common in females, accounting for 33%, 24%, 11% and 4% of the female cancers, respectively.

**Conclusions:**

Cancer incidence has remained relatively stable over time, but as shown elsewhere in sub-Saharan Africa the disease is a threat in The Gambia. The infection related cancers which are mostly preventable (HBV in men and HPV/HIV in women) were the most common. At the moment the data is not enough to detect an effect of hepatitis B vaccination on liver cancer incidence in The Gambia. However, we observed that monitoring case occurrence through PBCR is a key public health pre-requisite for rational planning and implementation of targeted interventions for improving the health of the population.

## Introduction

While the advent of the Acquired Immuno-Deficiency Syndrome (AIDS) contributed to renewed interest in AIDS-related or infection-related cancers and possible co-factors, there is still limited knowledge about cancer incidence and its evolution in relation to the rapid demographic and lifestyle changes in sub-Saharan Africa. The main reasons for this lack of knowledge are mainly due to the limited availability and access to programmes for cancer prevention, early diagnosis and treatment and the scarcity of population-based cancer registries with long periods of operation. Nevertheless, estimates of cancer occurrence suggest that cancer is a major public health concern in Africa and thus the need for development and implementation of rational cancer control programmes to curb the disease and its related effects. The undisputed basic and or expanded role that the PBCR can offer in such instances is well known [1-5].

Current literature show variations in the occurrence of cancer in sub-Saharan Africa and with the advent of the Human Immuno-deficiency Virus (HIV) infection and or AIDS pandemic the emergence of a new cancer pattern is eminent [6,7]. Currently in West Africa the most common cancers in men are liver and prostate and in women cervix and breast cancers [8-11]. In East and South of the continent, Kaposi sarcoma has now over shadowed the occurrence of oesophagus, cervix and liver cancer by emerging as the most common malignancy in both sexes in recent reports [6,12-14]. The continuous monitoring and evaluation of such information can provide for effective health planning and policy for cancer control in sub-Saharan Africa. This echoes the importance of establishing more population-based cancer registries in sub-Saharan Africa. In fact many reasons have been put forward to show why it is more of a necessity than a luxury to do so in developing countries [15].

The National Cancer Registry of The Gambia (NCR) was established in July 1986. Its main purpose is to provide for the final evaluation of The Gambia Hepatitis Intervention Study (GHIS) project. This desired role is being performed in conjunction with the basic role of a Population-Based-Cancer-Registry (PBCR) [16]. To our knowledge it is currently the only PBCR in sub-Saharan Africa that achieved nation-wide coverage including coverage of a substantial indigenous rural population. The main goal of the GHIS project is to evaluate the protective effectiveness of infant immunisation against chronic Hepatitis B Virus (HBV) infection in the prevention of chronic liver disease, specifically, primary liver cancer and cirrhosis later in adult life [17]. In principle therefore, the role of the NCR is more consistent with an expanded role as described for a PBCR (see reference [1]), than the basic role assumed by similar institutions in the context of sub-Saharan Africa [9,10,13,18-21]. Fulfilment of its basic role as a PBCR in the context of sub-Saharan Africa is evident from examination of its previous reports. These reports describe incidence of cancer during the period July 1986 – June 1988 [22], 1988-1997 [8] and an increase in female liver cancer with ethnic variations in the occurrence of cervix and breast cancer have been described recently [23]. In this study we utilised twenty years of data (1990-2009), to further characterise the baseline cancer incidence information collected as part of the GHIS project with an attempt to map out and demonstrate both the basic and expanded role assumed by the cancer registry, i.e. a PBCR in the context of hepatitis B and liver cancer control in sub-Saharan Africa.

## Methods

### Cancer records

The method of data collection and the problems encountered as well as the procedures of the cancer registry were described in previous reports [8,22]. Notification of cancer is voluntary in The Gambia and cancer registration is an active process. Trained cancer registry staff visits all health institutions, both public and private, engage clinicians to contribute to the register and scan the identified data sources for cancer diagnoses. Initially, the cancer registry operated from one base office, actively collecting data throughout the country. In 1997-1998, a decentralization process was developed to better integrate the operations of the cancer registry with the medical services. Trained cancer registration field staff were posted at the major tertiary care facilities, namely, Edward Francis Small Teaching Hospital (EFSTH) in Banjul, (formerly, Royal Victoria Teaching Hospital), Medical Research Council in Fajara, Armed Forces Provisional Ruling Council General Hospital in Farafenni and Bansang Hospital in Bansang, with responsibility to collect data on all cancer diagnoses within a specified geographical area of the country. The registry staff is also active in the co-ordination of an ultrasound examination service, channelling cytology and biopsy specimens to the only histopathology laboratory service based at the EFSTH, storing and managing the clinical files of cancer patients, and collecting blood and performing laboratory tests. Specifically, quantitation of alpha-fetoprotein and hepatitis B surface antigen (HbsAg) in plasma to assist clinical diagnosis of chronic liver disease mainly, primary liver cancer.

This study was conducted in The Gambia as part of the GHIS project which was approved by the Joint Gambia Government (GG) /Medical Research Council (MRC) Ethics committee. The study was endorsed by the local steering committee of the GHIS project. In line with most places elsewhere in the world, obtaining patient consent for inclusion of personal and tumour details, encrypted in the cancer registry database, was not required by any of the committees mentioned above.

The NCR database was automated from inception and is managed using the CANREG system [24] since the early ‘90s. It collects personal information including names, usual residence, age, sex, ethnic and civil status as well as details on the tumour including primary site, morphology, basis and date of diagnosis and hospital/clinic of initial diagnosis and registration. All patients diagnosed by a medically qualified doctor are eligible for inclusion into the database. Tumour site and morphology are coded according to the International Classification of Diseases for Oncology, third edition (ICDO-3) [25].

### Population data

**Figure 1:**
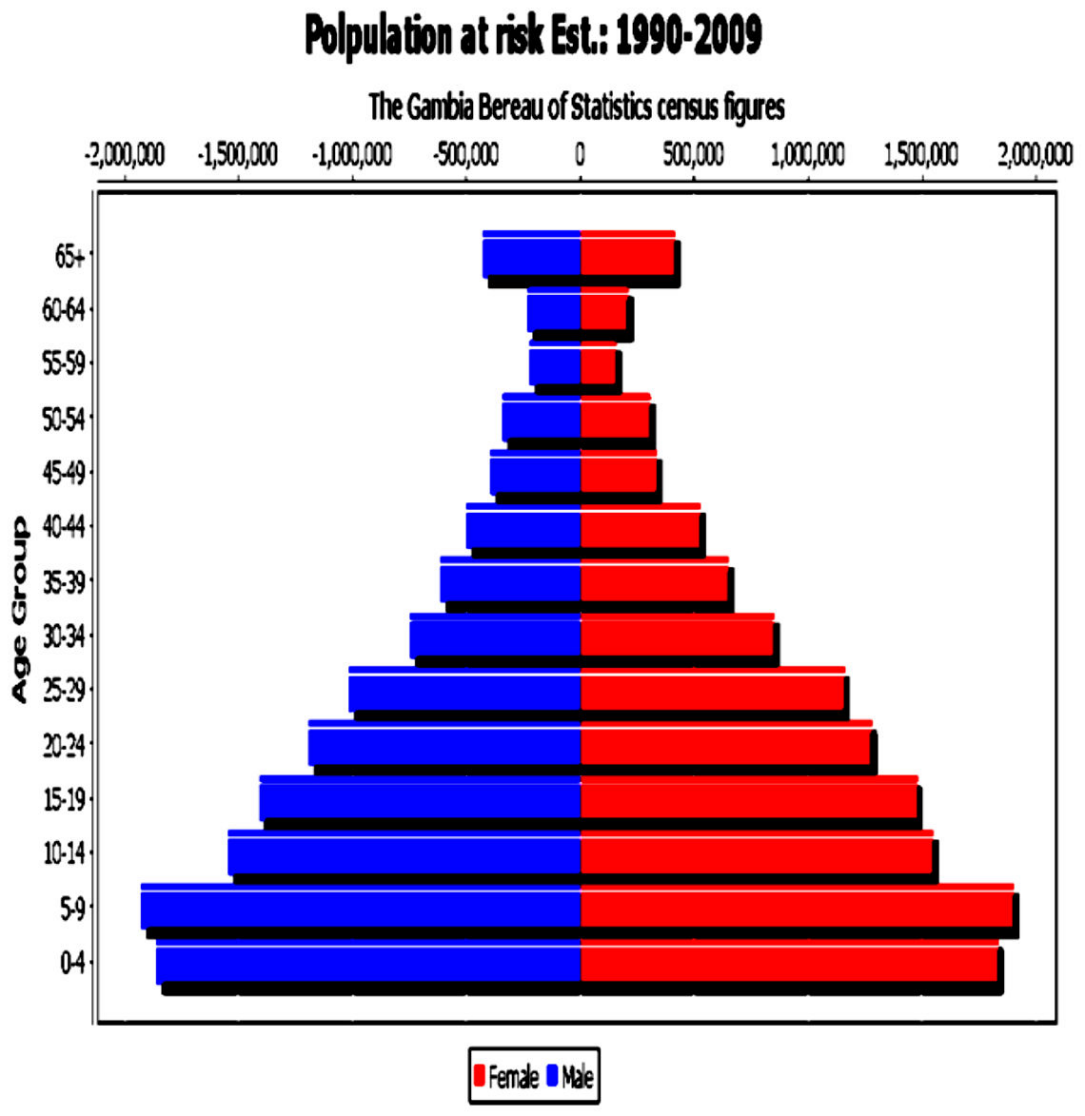
Estimated population at risk by age group and sex: The Gambia, 1990-2009.

Decennial population censuses have been performed in The Gambia since 1901. The population of The Gambia grew from 687,817 in 1983 to 1.4 million in 2003 (the latest officially published population census at the time of this analysis). Life expectancy at birth have also increased both in males and females to over 60 years in recent years [26]. In order to compute incidence rates and their 95% confidence intervals, we estimated the population at risk for each of the periods under review, using standard formula of population interpolation and extrapolation of the census figures of 1983, 1993 and 2003. Such that for period 1 and 2 the figures were interpolated using the census figures of 1983 and 1993, while the figures of period 3 and 4 where extrapolated using the 2003 census. Figure 1 shows the population pyramid of The Gambia for the period under review.

### Statistical methods

In this analysis we grouped data collected over a 20-year period, 1990-2009, into four by 5-year strata, namely, 1990-1994, 1995-1999, 2000-2004 and 2005-2009 (denoted as period 1, 2, 3 and 4, respectively in the text). For each of the four by 5-year periods and for the whole period under review and by five-year age groups, we computed the relative frequency (%) and crude incidence rates. The rates were adjusted for age using the world standard population [27] commonly utilised in the comparison of cancer registry information from different parts of the globe (see references [7,28]). Similarly, we calculated the corresponding 95% confidence intervals (95%CI) for each age-adjusted rate (ASR(W)) using the standard statistical formula described by Boyle & Parkin [29]. Differences in incidence between periods were tested using the Maentel-Haenzel method with modifications using the standard statistical formula described by Esteve and colleagues [30].

## Results

Over the 20-year period, i.e. 1990-2009, a total of 7,991 malignant tumours were registered among usual residents of The Gambia, 52% males and 48% females. In Tables 1 and 2, the number, relative frequency (%), ASR (W) with its corresponding 95%CI by primary cancer site and period, are shown for males and females, respectively. We show similar statistics in Table 3 for all cancer registrations by period and sex.

**Table 1:** Number of cases, ASR per 100,000 person-years with 95% Confidence Intervals of the most common cancers by primary site and period and all cancer registrations by period - Males: The Gambia, 1990-2009.

	**PRIMARY SITE OF CANCER (ICD-10)**				
**PERIOD**	Liver (C22)	Prostate (C61)	Lung+ Bronchus (C33-C34)	NHL (C82-C85)	Stomach (C16)
**1990-1994**					
No. of cases*(%)	*467 (61)*	*21 (3)*	*33 (4)*	*42 (5)*	*23 (3)*
**ASR (95%CIs)	29.0 (26.2-31.7)	1.7 (1.0-2.4)	2.5 (1.6-3.3)	2.0 (1.4-2.7)	1.5 (0.9-2.1)
**1995-1999**					
No. of cases*(%)	*551 (55)*	*43 (4)*	*43 (4)*	*56 (6)*	*26 (3)*
**ASR (95%CIs)	30.3 (27.7-33.0)	3.0 (2.1-4.0)	2.9 (2.0-3.8)	2.3 (1.6-3.0)	1.6 (1.0-2.3)
**2000-2004**					
No. of cases*(%)	*659 (61)*	*44 (4)*	*45 (4)*	*48 (4)*	*24 (2)*
**ASR (95%CIs)	31.4 (28.9-33.9)	2.9 (2.0-3.7)	2.7 (1.9-3.5)	1.4 (0.9-1.8)	1.3 (0.8-1.9)
**2005-2009**					
No. of cases*(%)	*809 (62)*	*85 (7)*	*37 (3)*	*68 (5)*	*37 (3)*
**ASR (95%CIs)	34.7 (32.1-37.2)	4.9 (3.8-5.9)	2.1 (1.4-2.8)	2.0 (1.5-2.5)	2.0 (1.3-2.6)
**Total (1990-2009)**					
No. of cases*(%)	*2,486 (60)*	*193 (5)*	*158 (4)*	*214 (5)*	*110 (3)*
**ASR (95%CIs)	31.6 (30.3-32.9)	3.2 (2.8-3.7)	2.5 (2.1-2.9)	%1.1 (1.6-2.2)	1.6 (1.3-1.9)

* (%) = Percentage of total cases in period; ** ASR = Age-standardized incidence rate per 100,000 person-years (Standardized to the World Standard population).

**Table 2:** Number of cases, ASR per 100,000 person-years with 95% Confidence Intervals of the most common cancers by primary site and period and all cancer registrations by period - Females: The Gambia, 1990-2009.

	**PRIMARY SITE OF CANCER (ICD-10)**			
**PERIOD**	Cervix uteri (C53)	Liver (C22)	Breast (C50)	NHL (C82-C85)
**1990-1994**				
No. of cases*(%)	*199 (31)*	*138 (21)*	*67 (10)*	*25 (4)*
**ASR (95%CIs)	13.9 (11.8-16.0)	9.6 (7.9-11.3)	5.0 (3.7-6.2)	1.2 (0.7-1.7)
**1995-1999**				
No. of cases*(%)	*361 (34)*	*205 (20)*	*100 (10)*	*36 (3)*
**ASR (95%CIs)	20.2 (17.9-22.4)	13.1 (11.2-15.1)	5.8 (4.6-7.0)	1.4 (0.8-1.9)
**2000-2004**				
No. of cases*(%)	*276 (30)*	*245 (26)*	*113 (12)*	*46 (5)*
**ASR (95%CIs)	13.7 (11.9-15.4)	12.2 (10.5-13.9)	5.5 (4.4-6.6)	1.6 (1.0-2.2)
**2005-2009**				
No. of cases*(%)	*418 (34)*	*337 (28)*	*156 (13)*	*39 (3)*
**ASR (95%CIs)	19.1 (17.2-21.1)	15.0 (13.3-16.8)	6.7 (5.5-7.8)	1.6 (1.0-2.1)
**Total (1990-2009)**				
No. of cases*(%)	*1,254 (33)*	*925 (24)*	*436 (11)*	*146 (4)*
**ASR (95%CIs)	16.9 (15.9-17.9)	12.8 (11.9-13.7)	5.8 (5.2-6.4)	1.5 (1.2-1.7)

* (%) = Percentage of total cases in period; ** ASR = Age-standardized incidence rate per 100,000 person-years (Standardized to the World Standard population).

**Table 3:** Number of cases, ASR per 100,000 person-years with 95% Confidence Intervals of the most common cancers by primary site and period and all cancer registrations by period - All cancer registrations: The Gambia, 1990-2009.

**PERIOD**	**Males**	**Females**
**1990-1994**		
No. of cases*(%)	*771 (100)*	*649 (100)*
**ASR (95%CIs)	47.8 (44.3-51.4)	44.7 (40.9-48.4)
**1995-1999**		
No. of cases*(%)	*994 (100)*	*1,049 (100)*
**ASR (95%CIs)	55.1 (51.5-58.7)	61.2 (57.2-65.3)
**2000-2004**		
No. of cases*(%)	*1,072 (100)*	*930 (100)*
**ASR (95%CIs)	51.3 (48.8-54.5)	45.4 (42.2-48.5)
**2005-2009**		
No. of cases*(%)	*1,305 (100)*	*1,221 (100)*
**ASR (95%CIs)	57.5 (54.2-60.8)	54.8 (51.5-58.1)
**Total (1990-2009)**		
No. of cases*(%)	*4,142 (100)*	*3,849 (100)*
**ASR (95%CIs)	53.3 (51.6-55.0)	51.8 (50.0-53.6)

* (%)=Percentage of total cases in period; ** ASR = Age-standardized incidence rate per 100,000 person-years (Standardized to the World Standard population).

In males, the most common cancers in each of the periods examined were liver, comprising 60% of all cancers, prostate (5%), lung and bronchus (4%), Non-Hodgkin Lymphoma (NHL) (5%) and stomach (3%). There was no significant increase in the evolution of incidence of any of the most common sites studied in males except for prostate cancer, which showed a doubling in incidence during period 4 compared to period 1. We did not observe any clear trend in the overall cancer incidence rates in females during the period examined here. Our analysis revealed that the most common cancers among female Gambians are cervix uteri which accounted for 33% of cancers in this group followed by liver cancer (24%), breast cancer (11%) and NHL (4%). Also, beside cervical cancer, the incidence rates of most major cancers shown in Table 1 and Table 2 for males and females respectively are comparable to those reported in a recent review of cancer incidence rates in West Africa [11]

### Histological diagnosis

Data quality, as may be judged from confirmation of clinical diagnosis of cases via histology and or cytology, has improved across the four periods which were examined in this analysis. Table 4 show the percentage of morphological verification of clinical diagnosis of cancer in The Gambia by site and period; and to show further details of this information, the percentage of the most common histological diagnosis by site were also provided (see Table 5). Overall, more than 20% of all cancer cases are now confirmed by histology in The Gambia and excluding liver, this figure reaches beyond 40%. Clinical diagnosis of liver cancer was less confirmed by histology, below 5% of cases after year 2000, coinciding with the period when recruitment of liver cancer cases for the second case-control study in The Gambia [31] was concluded. Indeed, the late presentation of liver cancer patients to hospital and the absence of treatment options after diagnosis make it medically and ethically not justified to carry out routine biopsies on these patients. This situation may change with the recent revival and expansion of the liver disease referral clinics in-country. The service is now run by two resident consultant Hepatologists involved with the GHIS project and an ancillary international research project in West Africa on treatment of chronic HBV infection in adults above age 29.

**Table 4:** Percentage (of total in period) of cancers morphology verified (MV %) by period and by site.

			**PERIOD**		
**PRIMARY SITE OF CANCER** (**ICD-10 code**)	**1990-1994**	**1995-1999**	**2000-2004**	**2005-2009**	**1990-2009**
Liver (C22)	5.0	3.8	4.1	0.9	**3.1**
Prostate (C61)	19.0	30.2	20.5	30.6	**26.9**
Lung + Bronchus (C33-C34)	18.4	10.0	9.8	16.7	**13.4**
NHL (C82-C85)	37.3	48.9	48.9	61.7	**50.6**
Stomach (C16)	8.7	28.6	18.2	46.8	**26.2**
Cervix (C53)	14.1	26.9	23.6	23.7	**23.0**
Breast (C50)	39.7	48.1	65.3	63.8	**56.9**
**All sites**	**16.7**	**24.5**	**23.9**	**24.1**	**22.8**

**Table 5:** Percentage (%) of the most common morphology diagnosis by primary site: The Gambia, 1990-2009.

**PRIMARY SITE OF CANCER** (**ICD-10 code**)	**MOST COMMON MORPHOLOGY** (**ICD-O-3 code**)	**%** (**NUMBERS**)
Liver (C22)	Hepatocellular carcinoma, NOS (8170)	82.1% (87/106)
Prostate (C61)	Small cell carcinoma, NOS (8041)	69.2% (36/52)
Lung + Bronchus (C33-C34)	Small cell carcinoma, NOS (8041)	12.0% (3/25)
	Squamous cell carcinoma, NOS (8070)	24.0% (6/25)
	Adenocarcinoma, NOS (8140)	12.0% (3/25)
NHL (C82-C85)	Malignant lymphoma, NOS (9590)	32.4% (59/182)
	Malignant lymphoma, non-Hodgkin, NOS (9591)	29.1% (53/182)
	Burkitt lymphoma, NOS (9687)	20.3% (37/182)
Stomach (C16)	Adenocarcinoma, NOS (8140)	50.0% (22/44)
Cervix (C53)	Squamous cell carcinoma, NOS (8070)	51.9% (150/289)
Breast (C50)	Infiltrating duct carcinoma, NOS (8500)	57.4% (147/256)

### Liver cancer

**Figure 2:**
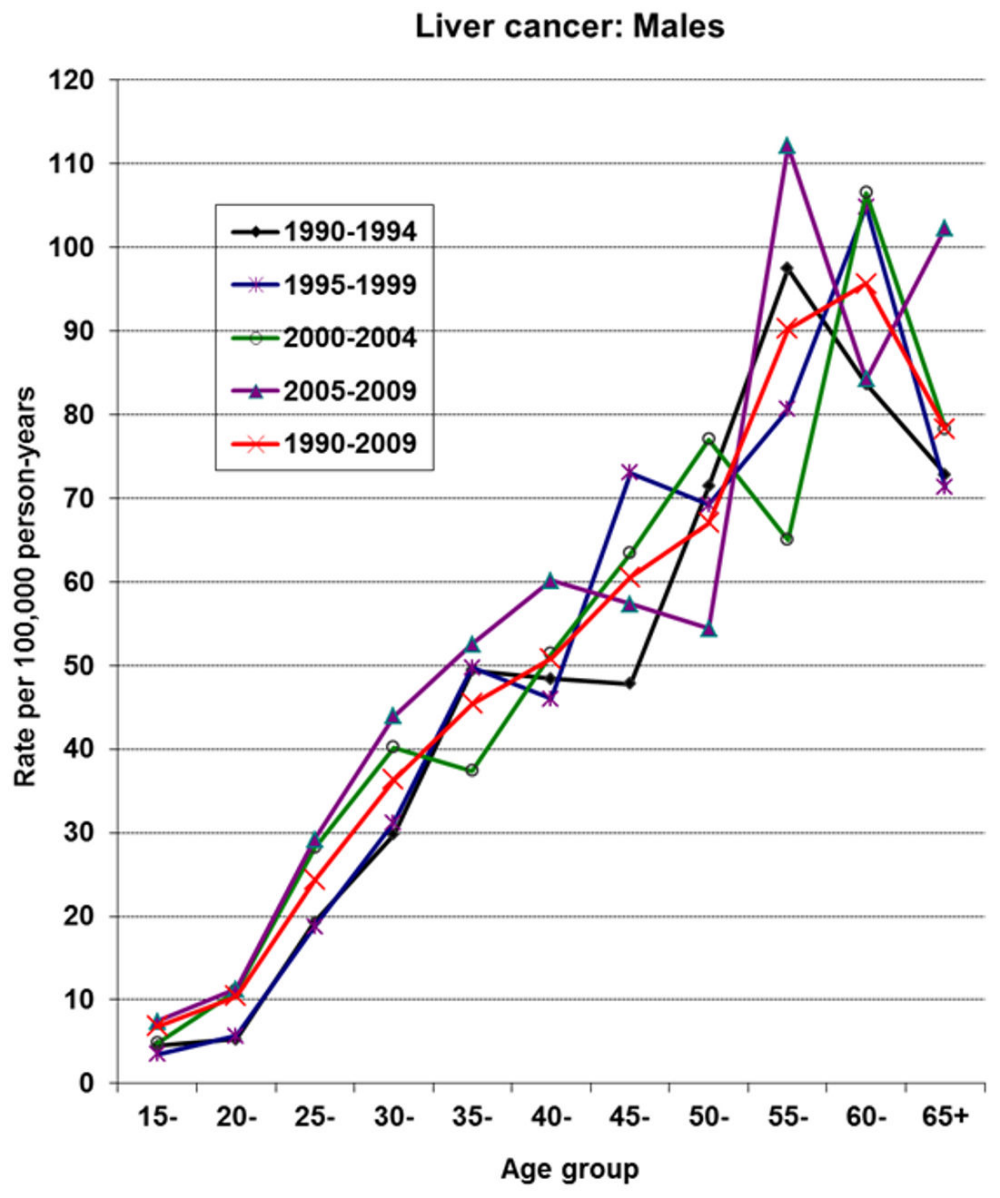
Age-specific incidence rates of liver cancer by period in male Gambians.

**Figure 3:**
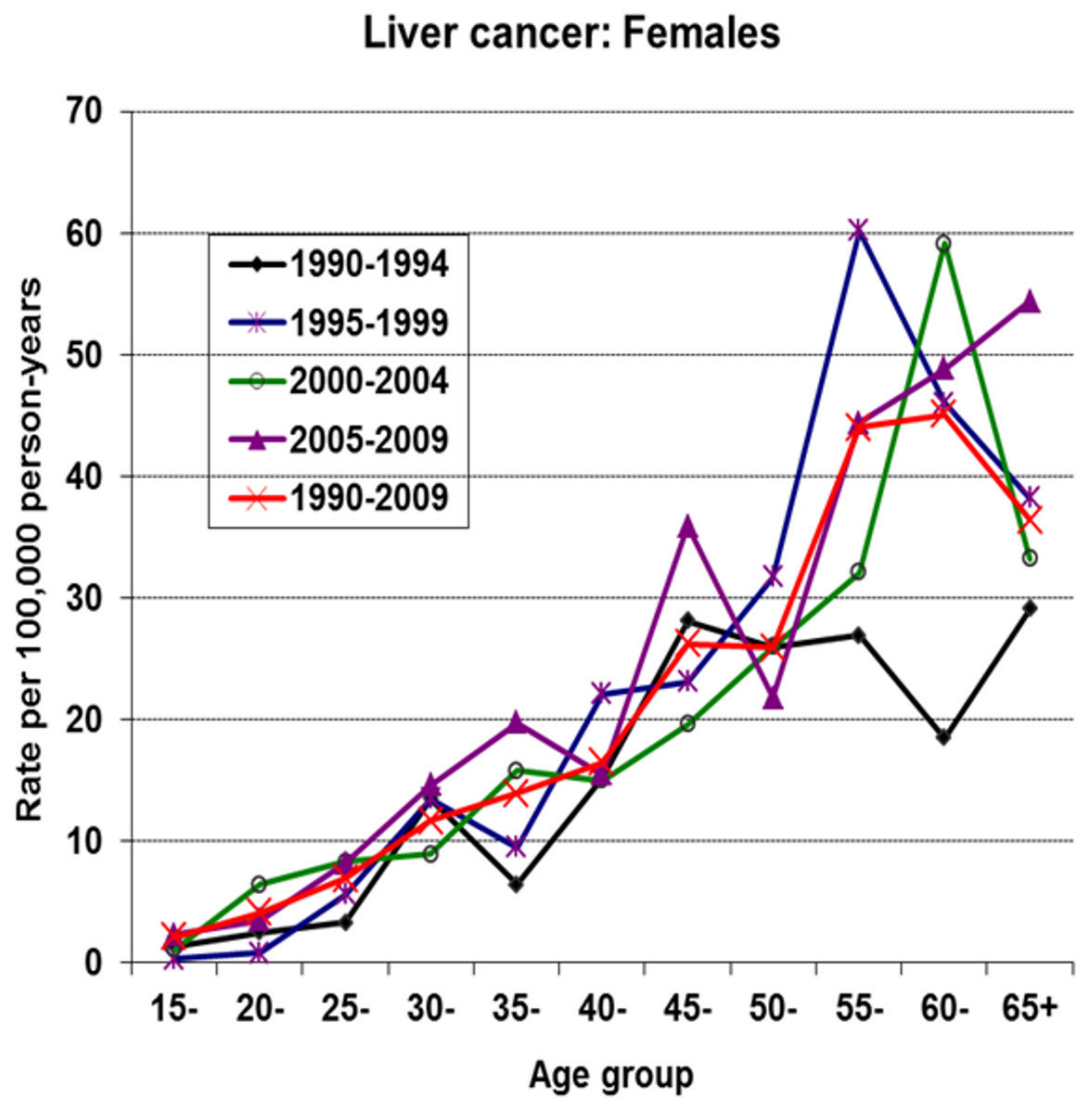
Age-specific incidence rates of liver cancer by period in female Gambians.

Figures 2 and 3 show the age-specific incidence curves of liver cancer by period in males and females, respectively. In each of the sexes, the peak of incidence shows a complex progressive advancement by age and period. Notably, there is a rapid rise in incidence rates in young male Gambians during all periods. This is followed by a display of incidence peaks after age 50 and a nadir in incidence observed in the oldest generation (i.e. beyond age 60). In contrast to female Gambians, a less dramatic rise in incidence rates in young ages is observed. A striking observation in recent times in females, is the shift in peak incidence from age group 50 in period 2 to age group 55 in period 3, followed by a continuous rise in incidence rates into the older ages during period 4. The shape of the incidence curve in period 4 is in contrast to the familiar pattern of a nadir in incidence in the oldest age range observed in these series from The Gambia. This observation may be suggesting improved case ascertainment in the NCR and or depicting a cohort effect in the older female generation, specifically above age 50. An earlier analysis of the NCR data covering the period 1988-2006 found the recent rise in female liver cancers in The Gambia to be statistically significant with a percentage change of 3.01 (95% CI [0.3-5.8]) [32].

### Cervical cancer

**Figure 4:**
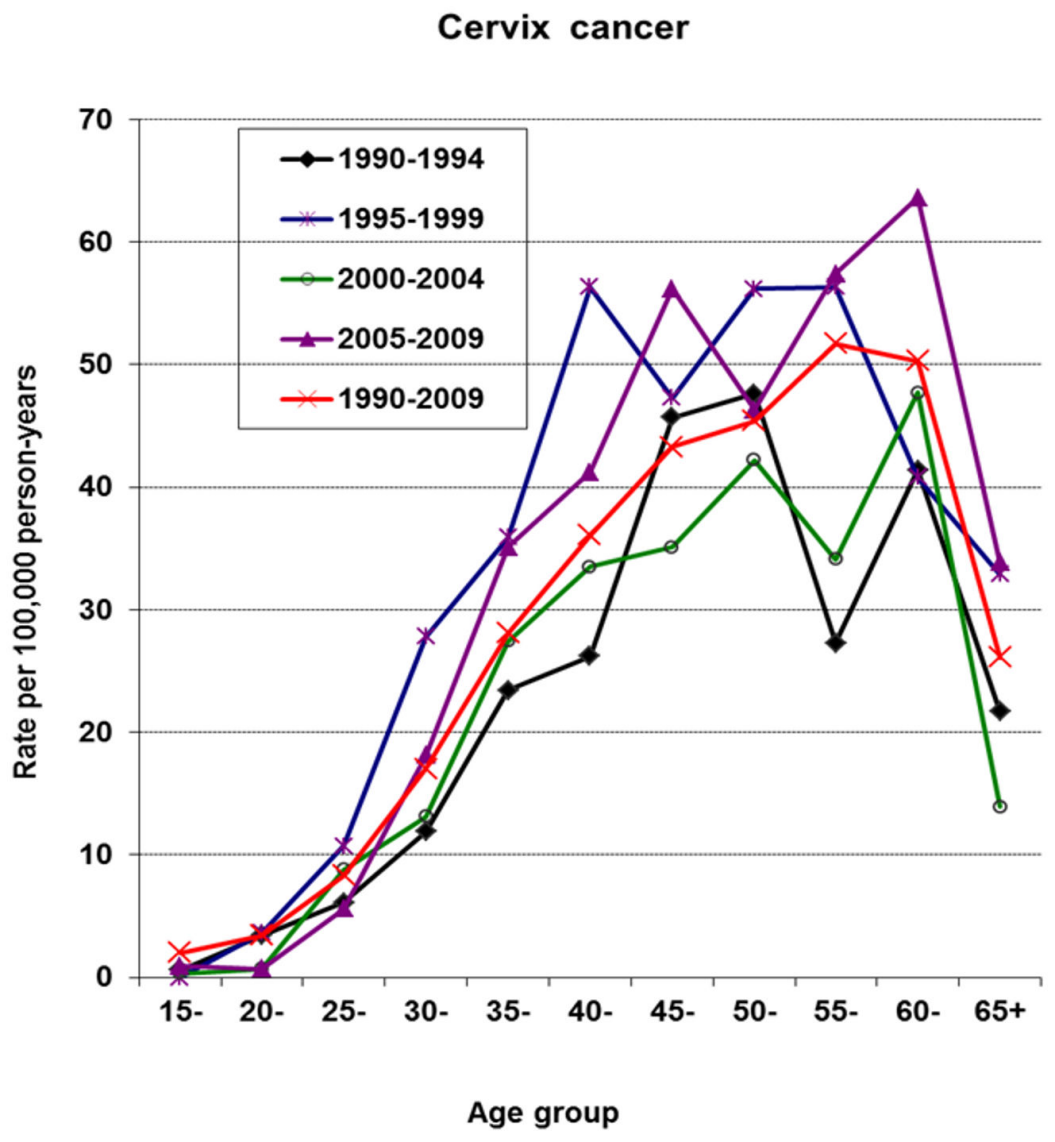
Age-specific incidence rates of cervix cancer by period.

In females, cervical cancer was the most common cancer across all periods. There is evidence of an increase in the diagnosis of the disease after period 1 and consequently, period 2 exhibits the highest incidence rate with ASR (W) at 20.1 per, 100,000 person-years. In period 4, the ASR (W) was 19.1 per 100,000 person-years. Figure 4 shows the age specific incidence curves by period. The rates show a steep rise in the youngest age groups and in addition a bi-modal pattern of occurrence after age 35. Although the incidence rates during the 1995-1999 period were by far the highest, the shape of the incidence curve shares similar features with those of the periods which follow.

### Prostate, lung plus bronchus, stomach, breast and NHL cancers

**Figure 5:**
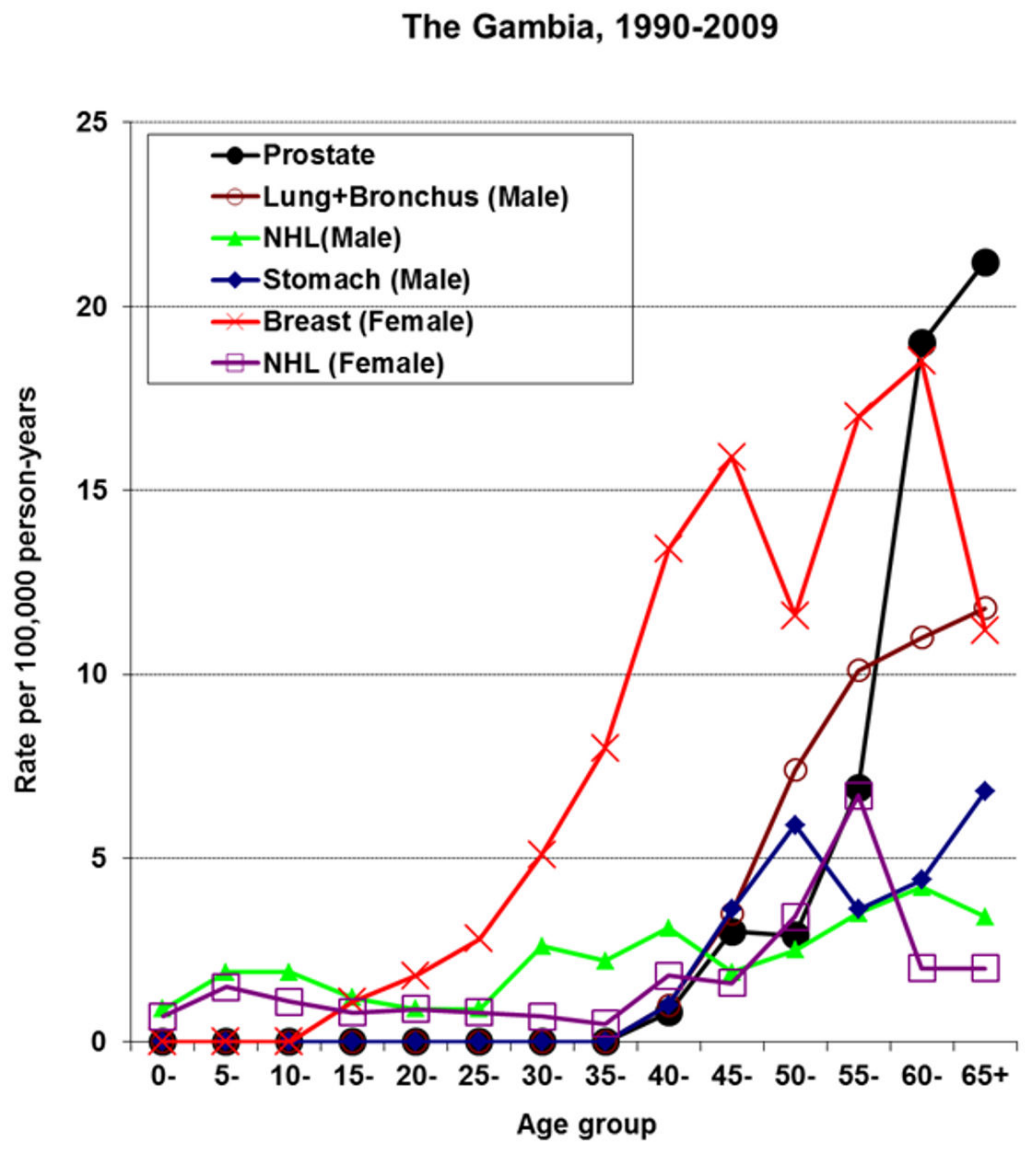
Age-specific incidence rates of other common cancers in Gambian Males and Females, 1990-2009.

The incidence curves of prostate, male lung plus bronchus cancers, male stomach cancers, female breast cancers and NHL in males and females are shown in Figure 5 for the period 1990-2009. During this 20-year period, in terms of the ASR(W)s, prostate, lung plus bronchus, NHL and stomach cancers were the second, third, fourth and fifth most common cancers in males. In females, breast and NHL were the third and fourth most common cancers during the period. A gradual increase in breast cancer incidence rates in young women reaching a peak at age 45 and exhibiting a second peak at age 55 followed by a sudden decline afterwards is shown in Figure 5. Such sharp decline in incidence rates mirrors the familiar phenomenon of under ascertainment incurred in the NCR which is particularly observed in the older age categories of this population. Furthermore, Figure 5 shows that female breast cancer is the most important cancer before age 55 and is exceeded only by prostate cancer after that age when it sharply declines in incidence. There is no breast cancer screening in The Gambia. The recent introduction of Prostate Specific Antigen (PSA) testing in The Gambia may have been responsible for the sharp upward turn of prostate cancer incidence after age 55 which continues to rise into older ages as expected, making it the most important cancer than lung plus bronchus and stomach cancers after age 50 in males. Although a doubling in the incidence rates of prostate cancer was observed during recent times in The Gambia (see Table 1) its occurrence is still dwarfed by the incidence rates reported elsewhere in West Africa [7].

NHL is the fourth most common cancer in male and female Gambians with Burkitt lymphoma (BL) being the most common morphological type in both sexes. In 1990-2009, 37 male BL cases (17% of male NHL cases) and 35 female BL cases (24% of female NHL cases) were registered. Majority of these cases, 92% and 87%, occurred below age 15 in males and females, respectively. Below age 15 NHL appears to exhibit a similar pattern of occurrence in both sexes with rates being higher in males than in females. However afterwards, especially in the older ages, a variable pattern in incidence curves is revealed with the females exhibiting a much higher incidence. Such that in females higher rates are observed after age 45 with a peak at age 55 and lower rates for women with age over 55. In males, the higher rates were observed from age 25 with peaks at age groups 30, 40 and 60 followed by lower rates at ages higher than 60. The difference and the various peaks in incidence rates observed in the older ages may be due an artefact of the small number of cases available to us in this analysis, again as a result of the under ascertainment observed in the older generation of this population [22].

## Discussion

Overall our analysis shows that the occurrence of the most common cancers in both sexes was relatively stable over time in The Gambia, with the exception of liver cancer in females and prostate cancer. Our assessment of cancer incidence further strengthens the evidence implicating the infection-related cancers of liver and cervix as the most important cancers in The Gambia. The potential impact of the current policy of immunisation of infants against hepatitis B infection on primary liver cancer incidence in The Gambia cannot be detected at this point in time. More data need to be collected overtime via the PBCR for that to be achieved by 2017-2020 (see reference [33]).

Previous analysis showed primary liver cancer as the most common cancer affecting male Gambians [8,22]. In this analysis the disease continues to be the leading cancer site across all periods in males (constituting 60% of all cancer cases in males) and in females it is the second most common cancer after cervix (constituting 24% of all cancer cases in females). While the age-standardised incidence rates suggest the disease is stable in male Gambians, in their female counterparts an increase was observed during recent years. Examination of the male: female ratios of liver cancer observed in The Gambia further elucidate this evidence. Specifically, the male: female ratios were 3:1 during the early 90s (i.e. period 1) stabilising at 2:1 during recent times, confirming the results of an earlier study (see reference [23]).

Case ascertainment in older ages was among the major concerns of cancer registration in the country from inception [22], but our recent analysis shows evidence suggesting improvements in case ascertainment, although mainly for liver cancer among the elderly population. Specifically, liver cancer in females has increased during the most recent period exhibiting an increased incidence rate into the older ages which is in contrast to the patterns observed in earlier periods. Also, the peak age of incidence of liver cancer by period, have shifted progressively more into the older ages in both sexes particularly in Gambian females. In females, the shift increased with age and period i.e. from age 55 in period 2 to above age 60 during recent times.

However, it is also possible that factors other than improvements in case ascertainment could be responsible for the increase observed in female liver cancer. These include factors associated with major lifestyle changes and viral factors, specifically, obesity and hepatitis C virus infection as described in an earlier review (see reference [23]). Moreover, it is now known that although the association between HBV infection and liver cancer is strongest below age 50 in The Gambia [34], above age 50, HCV infection is more common and contributes about 20% of HCC cases, particularly in the female population [31]. Thus, it follows that with the increase in life expectancy and improved completeness of cancer registration of the older generations, it is most probable that a growing number of female liver cancers will continue to be detected. This warrants a specific control strategy which includes screening for and treatment of HCV and HBV chronic infections to complement the current vaccination strategy against HBV. Such an approach may probably have greater impact on the occurrence of liver cancer in Gambian women. Currently, there is no public health policy for the screening and treatment of HBV and HCV chronic infections in The Gambia.

### Monitoring the final outcome of the GHIS project

GHIS is a long-term project, the final outcome of which will be evaluated via the national population-based cancer registration scheme established for the purpose i.e. the NCR. Details of the GHIS project have been described elsewhere [17]. The GHIS cohort is being followed-up via the NCR to identify all cases of hepatitis B-related primary liver cancer. These cases will be linked to the main GHIS immunisation database that was created from the beginning of the study. A probabilistic linkage approach is being developed for the purpose, in addition to efforts via the project, to specifically improve on the diagnosis of primary liver cancer in The Gambia. Currently, more than 90% of liver cancers diagnosed in-country are based on a positive ultrasound examination and or alpha-feto-protein test [22]. It has been reported that the use of ultrasound in combination with alpha-fetoprotein testing has specificity of above 90% in confirming clinical impression of liver cancer in the Sahel region [35]. This observation has been confirmed in a limited study on 36 Gambian patients for whom biopsies were available [36].

It is possible that our current observation in which liver cancer remains the most common cancer in male Gambians (more than half of the total number of registered cases), is due to the specific efforts being made by the GHIS project for the detection of the disease in the country as noted above. However, in contrast to female Gambians such a phenomenon was not observed. In this analysis, primary liver cancer is the second most common cancer (24%) after cervix uteri cancer which accounted for a third of all cancers in female Gambians.

Also evident in our current study is the demonstration of the level of consistency and robustness of liver cancer registration over the past two decades by the PBCR as achieved in the context of the GHIS project. In fact, in a recent study the evidence of high completeness of liver cancer registration amongst young Gambian male adults, the key target population for monitoring the final outcome of the GHIS project, was reported [37]. In this analysis, we observed a much higher risk of liver cancer than assumed for the country at the time of designing the GHIS project (see reference [17]). Such information has bearing on the long-term monitoring and evaluation of the final outcome of the GHIS project. Hepatitis B vaccination was successfully integrated into policy in The Gambia since February 1990 and has reduced infection rates in children to below 0.5%. This finding suggests a future decline in the incidence of Primary Hepatocellular Carcinoma (PHC) in The Gambia. Therefore, with the proper utilisation of information provided via the PBCR from now on, it will be possible to estimate and validate reports on the time to conclusion of the GHIS project. Current estimates along those lines, suggest that enough cases of PHC for the institution of proper analysis for detection of a statistically significant effect of the intervention (i.e. at the 5% level of statistical significance) will become available in The Gambia from 2017 [33]. If such an analysis is accomplished the information provided will be key to rationale health planning and policy in The Gambia and elsewhere in sub-Saharan Africa. Ensuring the provision of such key information is testimony to the expanded role being fulfilled by the PBCR in The Gambia. Thus, irrespective of the quality issues listed for the PBCR in The Gambia, specifically poor ascertainment in the rural areas, older ages as described earlier (see reference [22]) and during recent times (see reference [37]), overall the evidence shows that the registry will indeed serve as an effective tool for the evaluation of the GHIS strategy and continue to fulfil its expanded role as stated above.

### Monitoring the occurrence of the other common cancers

To accomplish its basic function as a PBCR in the context of sub-Saharan Africa the NCR monitors the occurrence of all other cancers in addition to liver cancer. In this analysis we observed that cervical cancer is the most common cancer in The Gambia after liver cancer in males, constituting 33% of all cancers in females. This compared to the figures for all Africa where it accounted for 23% of all cancers in females (see reference [11]), makes its relative weight of occurrence in The Gambia much higher than elsewhere in the continent. Over a period of two decades, we found the incidence of cervix cancer to vary between 13.7 and 20.2 in The Gambia. Though there is a risk of under-estimation in The Gambia [8], it should be considered that the excess of incidence in other countries of sub-Saharan Africa may be attributable to the higher prevalence of HIV. The prevalence of HIV infection is still low in The Gambia, below 2% [38,39]. Indeed HIV-infected heterosexual women carry a 6-fold risk of developing cervix cancer than the general population [40]. However it is important to note that relative risks of AIDS-associated tumours, including cervical cancer are lower in Africa than those reported in western countries [41] but it is difficult to say whether this is attributable to delayed access to antiretroviral and competing causes of AIDS-related deaths. Nevertheless, in The Gambia the evidence suggest under-reporting of the disease particularly among the older women. Close examination of the incidence rates of cervix uteri suggests better case ascertainment of the disease below the age of 55 in recent times. However, under ascertainment cannot be ruled out above this age group. Elsewhere in sub-Saharan Africa much higher rates of cervix uteri cancer have been reported [7]. The rapid decline in incidence rates exhibited in the older age groups may be responsible for the difference in cervix cancer incidence observed in The Gambia compared to other low resource areas of the globe [7]. Also, there is currently no organised screening or other prevention programme against cervix cancer in The Gambia that could significantly improve case ascertainment. Moreover, in The Gambia to what extent older women seek care for this disease or other common cancer is not documented. It is possible such patients would rather choose to die at home than present at hospital with terminal disease, particularly, in the absence of adequate palliative care in-country.

Breast cancer was among the top four cancers in Gambian women being the most important after female liver cancer. In this analysis we observed that most breast cancer diagnoses were in pre-menopausal women. This is a key characteristic of the occurrence of this disease in sub-Saharan Africa and among women of African origin resident elsewhere in the developed world [42,43]. Furthermore, the Gambia exhibits significant ethnic variations in female breast cancer incidence according to recent analysis [23]. The observation of such interesting features of cancer incidence in this part of the globe should warrant the conduct of collaborative analytical studies to further elucidate the genetic and environmental determinants of the disease. Such efforts can lead to more effective public health strategies to combat breast cancer globally.

Prostate cancer occurrence has doubled overtime and exhibits a sharp increase in incidence after age 50. However, the incidence of the disease is still very low in The Gambia compared to elsewhere in West Africa, specifically, in Nigeria were it has increased in incidence over time to become the leading cancer in men and forming 11% of all male cancers [44]. Organised screening has been implicated as the cause of increases in prostate cancer incidence elsewhere in the developed countries, especially among African-Americans [45]. The pattern and evolution of prostate cancer incidence due to the effects of introduction of screening organised or opportunistic, and improved cancer registration, remains to be elucidated in low resourced countries. In The Gambia, the increase seen in prostate cancer may be due to an artefact, specifically, improvement in case ascertainment in the PBCR as a result of the recent availability and increased utilisation of serum prostate-specific antigen (PSA) testing in the country.

It is interesting to note that apart from cervix uteri, female breast and prostate cancer, the incidence rates of which differ from elsewhere in West Africa, all the other common cancers studied here had similar rates of occurrence. Specifically, the corresponding ASR(W)s of liver, lung plus bronchus and NHL were comparable to elsewhere in West Africa [7]. This may be suggesting that the inherent under-ascertainment of cancer cases via population-based cancer registration may not be adversely different in West Africa.

## Conclusions

To our knowledge, we have conducted one of the longest assessments of cancer incidence in sub-Saharan Africa based on continuous population-based cancer registration covering an indigenous African population largely in rural dwelling. Current reviews of cancer in Africa show cervix, breast, and HIV-associated Kaposi’s sarcoma as the most common malignancies among indigenous Africans. In male Africans, the currently reported top five cancers were: Kaposi’s sarcoma (KS) (forming 13% of all cancers in males) and cancer of the liver (15%), prostate (10%), bladder (6%), and NHL (6%), while in their female counterparts these were, cervix cancer (which makes up 23% of all cancers in females), breast cancer (19%), Kaposi’s sarcoma (5%), liver cancer (5%) and NHL (4%) (See reference [11]). Our current analysis includes all of the above top five cancers but KS and bladder. In contrast, we show that in The Gambia, lung plus bronchus and stomach cancers are also among the top five. Such observations are indications that there are inherent differences in the cancer profile between the various populations and or regions of the continent. There is therefore the need to elucidate these differences in order to assist the formulation and or adaptation of appropriate cancer control policies to suite the various populations of the continent. The current series from The Gambia adds information to the existing knowledge of cancer incidence in sub-Saharan Africa. In principle, the information adds clarity and precision to designing, implementing and evaluating targeted interventions aimed at reducing the burden of the most common cancers in the continent and health improvement for the population.
